# Experimental and Numerical Study of Coupled Metronomes on a Floating Platform

**DOI:** 10.3390/e27090908

**Published:** 2025-08-27

**Authors:** Xiaolongzi Wu, Caiyi Zheng, Zhao Lei, Yu Qian, Zengru Di, Xiaohua Cui

**Affiliations:** 1 School of Systems Science, Beijing Normal University, Beijing 100875, China; 202331250004@mail.bnu.edu.cn (X.W.); zdi@bnu.edu.cn (Z.D.); 2 Department of Physics, Beijing Normal University (Zhuhai), Zhuhai 519087, China; 202211079182@mail.bnu.edu.cn; 3 College of Physics and Optoelectronic Technology, Baoji University of Arts and Sciences, Baoji 721007, China; leizhao1010@163.com (Z.L.); qianyu0272@163.com (Y.Q.); 4 International Academic Center of Complex Systems, Beijing Normal University, Zhuhai 519087, China

**Keywords:** synchronization, coupled oscillators, metronomes, nonlinear dynamics

## Abstract

We investigated synchronization behavior using an experimental setup consisting of two metronomes placed on a platform floating over water. By setting the metronomes to oscillate perpendicular to the line between them, we observed three distinct modes of movement: in-phase synchronization, anti-phase synchronization, and synchronization with a fixed phase difference. While this last mode resembles phase-locking, it is important to distinguish that phase-locking typically refers to an oscillator’s response to external pacing, whereas the fixed phase difference observed in our study emerges from the mutual interaction between two metronomes. The frequencies of oscillations, and the placement of the metronomes are also changed to check the reliability of the new phenomenon. Even if we changed the material of the platform to a heavier one or turned around one of the metronomes, synchronization with a fixed time delay still was still observed. Drawing on previous research, we developed mathematical equations to model the coupled metronomes and performed numerical simulations that successfully reproduced all three observed phenomena. The simulation results showed excellent agreement with our experimental observations. These findings contribute to our understanding of coupled oscillators and may have potential applications in various fields.

The “synchronization of clocks” that Huygens discovered more than 300 years ago continues to attract significant research attention, as oscillations pervade both the physical world and living systems, manifesting in phenomena ranging from mechanical vibrations to biological rhythms. With advances in experimental methods and computational technology, pendulum studies have become increasingly sophisticated. Experimentally, researchers commonly use metronomes to simulate single pendulums, and the study of multiple metronomes has emerged as a prominent research focus in the past decade. Two metronomes can oscillate in-phase or anti-phase, while multiple metronomes can even display chimera states. In this paper, we investigate two metronomes with close frequencies placed on a platform floating over water and examine their synchronization behavior. The metronomes can oscillate in phase, anti-phase, and in a third kind of synchronization. Specifically, unlike Huygens’ pendulums, one metronome can delay another with a fixed time difference, which we term “synchronization with a fixed phase difference.” Moreover, the time differences can vary by changing the initial conditions. This phenomenon resembles phase-locking in pulse-coupled oscillators, though here synchronization emerges from persistent metronome interactions. We explore the underlying mechanisms of these phenomena through experimental investigation and theoretical modeling. Our numerical simulations corroborate these experimental results. More broadly, our findings advance the understanding of coupled oscillator dynamics and may have implications for maritime engineering applications.

## 1. Introduction

The study of coupled metronomes represents a convergence of historical discovery, natural phenomena, and practical applications, encompassing a narrative that spans centuries and crosses multiple disciplines. From Christian Huygens’ seminal observation of pendulum clock synchronization in 1665 to contemporary investigations of mechanical and quantum system dynamics, the study of synchronization through coupled metronomes provides unique insights into interconnected systems [1,2,3,4,5]. Huygens’ pioneering work established the foundation for understanding system harmonization, a concept that has since been extensively explored across various scientific fields. These include real pendulum experiments [6] or theoretical discussion [7], synchronization control [8], theoretical nonlinear oscillators [9,10], even liquid crystalline oscillators [11], and oscillations in neuroscience [12], engineering [13], and so on.

In 2002, Pantaleone pioneered the study of coupled metronomes on a moving platform [14]. Since then, research in this field has expanded significantly, revealing diverse phenomena. Pantaleone demonstrated synchronization between two coupled metronomes both experimentally and analytically. Using similar apparatus, Ulrichs et al. provided numerical results showing synchronization onset in systems of 2, 3, and 100 globally coupled metronomes [15]. Kapitaniak et al. comprehensively synthesized research on coupled metronomes, providing systematic analyses and numerical results of their dynamical behavior [16,17,18]. The following work encompassed various aspects, including connector stiffness effects on system states [19], dynamics of rotating pendulums on movable beams [20], irrational phase synchronization [21], in-phase and anti-phase synchronization on moving platforms [22,23], and three-metronome coupling dynamics [24]. More recently, Strogatz et al. conducted detailed investigations into movement types and theoretical models of coupled metronomes [25,26]. Their research revealed that platform friction, previously modeled as velocity-proportional, should follow Coulomb-type behavior. These mechanical oscillators, when coupled, demonstrate complex behaviors including in-phase and anti-phase synchronization, illustrating the intricate interplay of forces governing synchronized states in coupled oscillatory systems.

In this study, we investigate a system of coupled metronomes placed on a floating platform, with the metronomes aligned along the platform’s long axis while their oscillations occur perpendicular to this axis. This study specifically examines how the mutual interaction between the two metronomes affects their behavior during the vertical oscillations. Our experiments reveal three distinct types of synchronization: in-phase synchronization, anti-phase synchronization, and synchronization with a fixed phase difference. The last phenomenon, which we term fixed-phase-difference synchronization, represents a novel observation in coupled metronome systems. We employ video recording to track metronome trajectories and develop an identification program to analyze their motion patterns. Furthermore, we construct a theoretical model to analyze the system dynamics and perform computer simulations. Our simulations demonstrate excellent agreement with the experimental observations.

## 2. Experimental Setup and Measurement Methods

**Figure 1:**
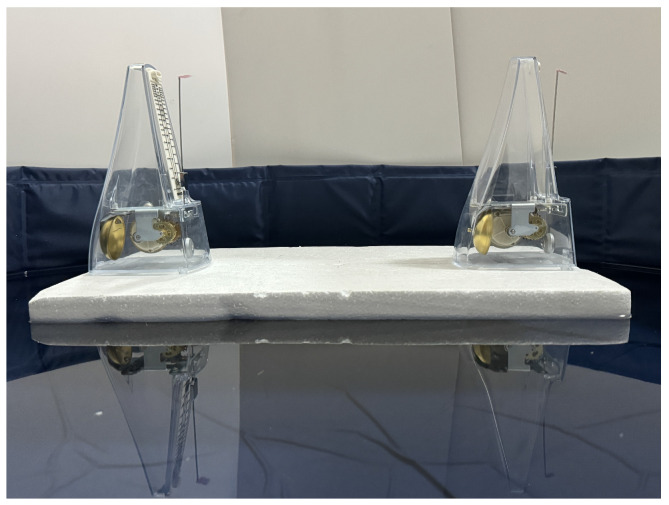
The setup of our experiment, consisting of two metronome parts and one platform. The whole setup is floating over the water.

The experimental setup comprises two metronomes mounted on a platform floating over water, as shown in Figure 1. The platform can move freely in two dimensions on the water surface. The metronomes are positioned along the platform’s long axis, with their oscillations occurring perpendicular to this axis. This configuration differs from previous coupled metronome experiments in two key aspects: the oscillation direction is perpendicular to the long axis, and the platform exhibits two-dimensional movement while floating on water, being weakly driven by the pendulum oscillations of the metronomes.

For this study, we selected Wittner’s Super-Mini-Taktell metronomes (Series 890, weighing 94 g) for their lightweight construction, cost-effectiveness, and accessibility. These metronomes operate via a manually wound spring mechanism. Their frequency can be adjusted by modifying the position of a weight on the pendulum’s bob, effectively altering the pendulum’s length, while these metronomes typically operate within a range of 40 ticks per minute (largo) to 208 ticks per minute (prestissimo), they are not limited to this scale. In our experiments, the metronomes were set to the andante setting, achieving 78 ticks per minute, corresponding to a period of approximately 0.77 s. The floating platform is constructed from polystyrene with a density of approximately 0.008 g/$m^{3}$.

The experiments are recorded with a camera and a custom identification program is developed for motion analysis. The program identifies differently colored markers placed at the balance position and tips of the metronomes. It calculates the distances ($d_{1}$,$d_{2}$) between each tip and its corresponding balance position, i.e., the amplitude of vibration. These swing angles are given by

(1) $$\phi_{i}=\arcsin\frac{d_{i}}{l^{\prime }},\left(i=1,2\right)$$

where $l^{\prime }$ represents the pendulum length. In the experiment, we varied the initial conditions of two metronomes and observed their motion. After multiple trials, we summarized the results of their movement.

## 3. Experimental Results and Analysis

To investigate the dynamics of coupled metronomes on a floating platform, we conducted experiments using an identification program. With randomly set initial states of the metronomes, we observed three distinct phenomena: in-phase synchronization, anti-phase synchronization, and synchronization with fixed phase delay.

**Figure 2:**
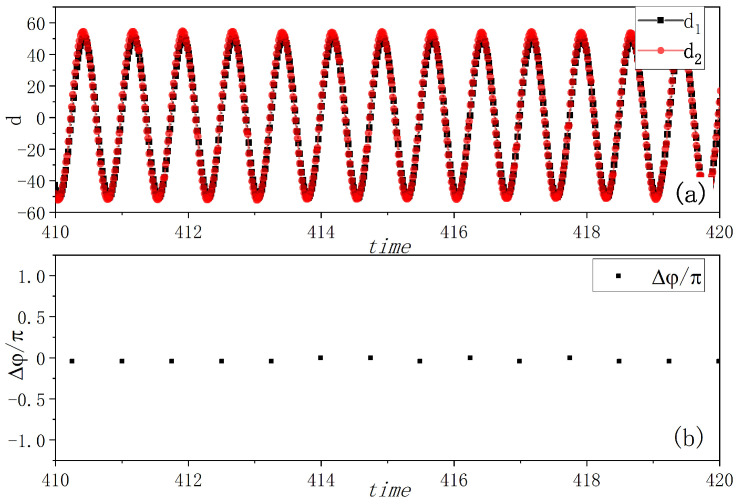
Experimental in-phase synchronization of the coupled metronomes. (**a**) The distances between tips of metronomes and the balance positions $d_{i}\left(i=1,2\right)$, i.e., the amplitudes of vibrations, are plotted by time. The movements of the two metronome pendulums are fully synchronized. (**b**) The phase differences Δφ of the two metronomes are calculated at the time when one metronome arrives at the balance position. They are nearly zero as plotted, which align with the phenomenon in (**a**).

Figure 2 illustrates the results of in-phase synchronization. The pendulum tips’ movements were captured by camera and analyzed using a computer program. The distances between the tips and their corresponding balance positions, i.e., the amplitudes of the vibrations $d_{i}\left(i=1,2\right)$, are plotted in Figure 2a, which clearly shows the synchronous movement. For analytical purposes, the angle of inclination $\phi_{i}\left(i=1,2\right)$ is converted to phase of swing $\varphi_{i}$, where the phase $\varphi_{i}$ is given by

(2) $$\varphi_{i}=\arccos\left(\frac{\phi_{i}}{\phi_{i}^{\max}}\right),i=1,2$$

with $\varphi_{i}$ being 0 or π at the point of maximum swing, while π/2 or −π/2 at the point of balance position. $\phi_{i}^{\max}$ is the highest angle of inclination, (i=1,2). The phase difference $\Delta \varphi =\varphi_{1}-\varphi_{2}$ between the two metronomes, calculated at the time when one metronome is at the balance position, shown in Figure 2b, remains approximately zero. This implies that the two metronomes have achieved synchronization.

**Figure 3:**
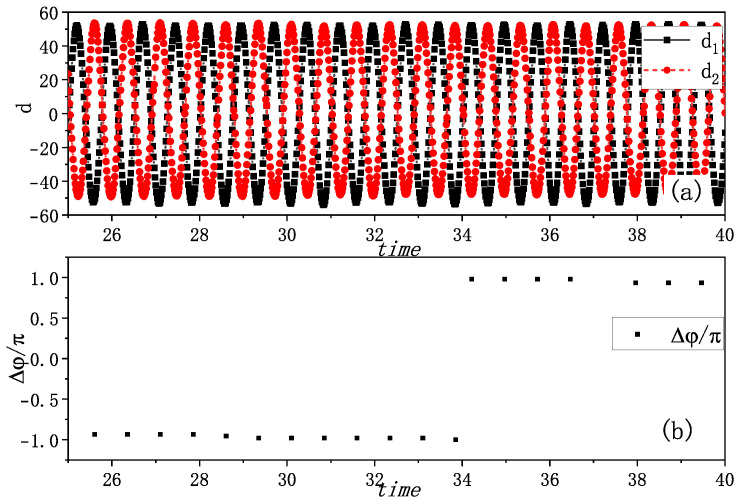
Experimental anti-phase synchronization of the coupled metronomes. (**a**) The distances between tips of metronomes and the balance positions $d_{i}\left(i=1,2\right)$, i.e., the amplitudes of vibrations, are plotted by time. The two tips move oppositely and show an anti-phase synchronization. (**b**) The phase differences Δφ of two metronomes are calculated at the time when one metronome arrives at the balance position. They are nearly π or −π as plotted, which align with the phenomenon in (**a**).

Anti-phase synchronization emerged under different initial conditions, characterized by a phase difference of π or −π. Figure 3a shows the amplitudes of vibrations of the two metronomes $d_{i}\left(i=1,2\right)$, showing their opposing oscillation patterns. The phase differences $\Delta \varphi =\varphi_{1}-\varphi_{2}$, calculated at the time when one metronome arrives at the balance position, are plotted in Figure 3b, and maintain a value close to π (or −π). This suggests that the two metronomes are oscillating in opposite phases, exhibiting anti-phase synchronization.

**Figure 4:**
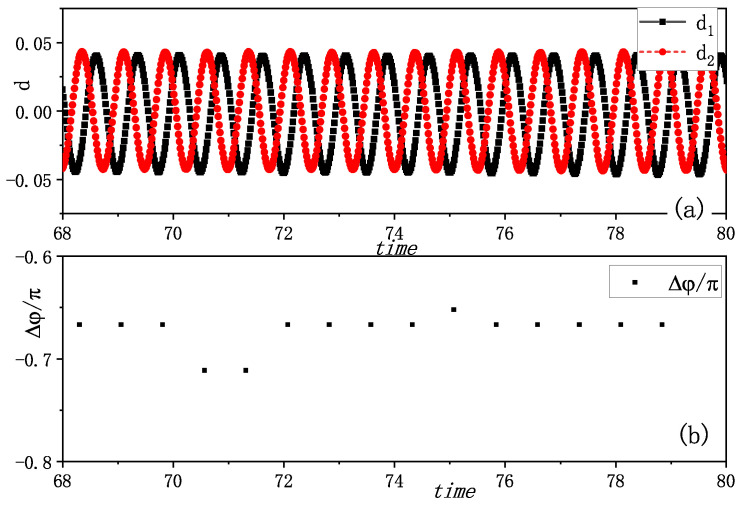
Experimental synchronization with a fixed time delay of the coupled metronomes. (**a**) The distances between tips of metronomes and the balance positions $d_{i}\left(i=1,2\right)$, i.e., the amplitudes of vibrations, are plotted by time. One metronome moves ahead of the other one, and the time differences between the maximum swing position seems a fixed value. (**b**) The phase differences Δφ of two metronomes are calculated at the time when one metronome arrives at the balance position. They are nearly −0.666π, and it means that the two metronomes oscillate with a fixed phase difference. We call this phenomenon as synchronization with a fixed phase difference. It is a new phenomenon, which is similar to phase-locking in pulse-coupled oscillators, while here, this kind of synchronization appears from persistent interaction of two metronomes.

A third interesting phenomenon emerged when varying the initial states: synchronization with constant phase difference. Figure 4a shows the two amplitudes of vibrations exhibiting a distinct time delay, and the time delay looks like a constant. We also calculated the phase differences Δφ of two metronomes at the time when one metronome arrives at the balance position, and found the phase differences converging to −0.666π (shown in Figure 4b). We verified this behavior through multiple experimental trials, consistently observing this time-delayed synchronization with various constant phase differences. Additional evidence is presented in Supplementary Materials Figure S1, showing another example of the revolutions of vibration amplitudes and the corresponding phase difference.

We also varied the vibrational frequency to observe the synchronization, and again observed the phenomenon of fixed-phase-difference synchronization. One of the corresponding results are placed in Figure S2 of the Supplementary Material. These synchronization patterns were maintained if one of the metronomes in Figure 1 rotated π, making the two metronomes vibrate back to back or face to face. Moreover, we changed the material of the platform to thermoplastic elastomer (TPE, $0.121\;g/m^{3}$), all three types persisted. These results demonstrated that the third synchronization is robustness.

Our experimental investigation of coupled metronomes reveals that the observed synchronization phenomena, particularly the “synchronization with a fixed phase difference” regime, are fundamentally governed by interaction dynamics rather than intrinsic oscillator properties. In the idealized case of identical intrinsic frequencies, the system establishes persistent synchronization with a fixed phase difference, where the specific phase offset is determined by initial conditions and remains stable over time. However, under realistic experimental conditions where intrinsic frequencies exhibit inevitable manufacturing variations, we observe that the maintenance of fixed-phase-difference synchronization is entirely mediated by interaction through the platform.

## 4. Theoretical Model

According to the model used in [16,22], and based on Euler–Lagrange equations, the governing equations for the motion of pendulums and the platform are

(3a)mil2ϕ¨i+mix¨cosθ+y¨sinθ+riθ¨lcosϕi+cϕiϕ˙i+miglsinϕi=MD,i=1,2(3b)M+∑i=12mix¨+cxx˙+∑i=12milϕ¨icosϕi−ϕ˙i2sinϕicosθ=0(3c)M+∑i=12miy¨+cxy˙+∑i=12milϕ¨icosϕi−ϕ˙i2sinϕisinθ=0(3d)112Mb2+∑i=12miri2θ¨+cθθ˙+∑i=12rimilϕ¨icosϕi−ϕ˙i2sinϕi=0

where $M_{D}$ is the momentum supplied by the escapement mechanism,

(4) $$M_{D}=\left\{\begin{array}{cc} 0.0075, & \mathrm{if}\;\dot{\phi }>0\;\mathrm{and}\;0<\phi <\frac{\pi }{36}\\ -0.0075, & \mathrm{if}\;\dot{\phi }<0\;\mathrm{and}\;-\frac{\pi }{36}<\phi <0\\ 0, & \mathrm{otherwise} \end{array}\right.$$

**Figure 5:**
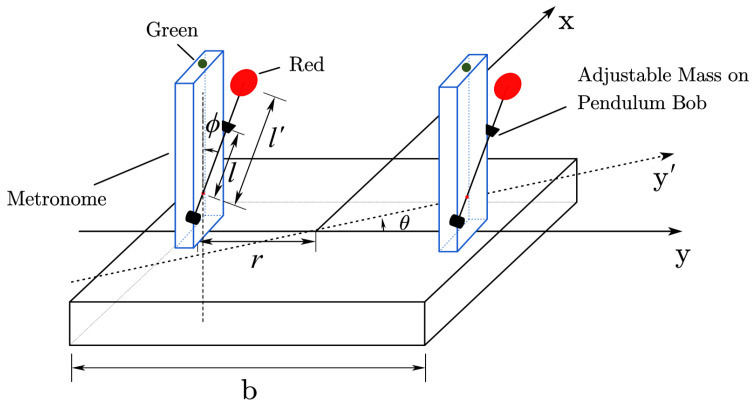
Scheme of experimental setup. The platform can move over the water, The variables *x* and *y* represent its vertical and horizontal displacements of the platform, respectively. The θ is the rotating angles of the platform. *r* is the distance between the metronome and the center of the platform, *b* is the length of the platform, and $\phi_{i},i=1,2$ denote the swing angles of the metronomes. *l* is the distance between the weight and the fixed point of the pendulum, while $l^{\prime }$ is the distance between the tip and the fixed point of the pendulum.

Here, as shown in Figure 5, *x* and *y* denote the vertical and horizontal displacements of the platform, respectively, with corresponding velocities $\dot{x}$, $\dot{y}$ and accelerations $\ddot{x}$, $\ddot{y}$. The rotational angle of the platform is denoted by θ. The drag coefficients associated with the platform motion on water are $c_{x}$ and $c_{y}$ (with $c_{x}=c_{y}$), while $c_{\theta }$ represents the rotational drag coefficient. Each metronome has a mass $m_{i}$ and a pendulum length *l* (measured from the adjustable bob to the rotation point). The angular displacement, angular velocity, and angular acceleration of the *i*-th metronome are denoted by $\phi_{i}$, ${\dot{\phi }}_{i}$, and ${\ddot{\phi }}_{i}$ (i=1,2), respectively. The coefficient $c_{\phi_{i}}$ characterizes the damping due to viscous friction. The driving torque $M_{D}$, provided by the escapement mechanism, supplies the energy required to overcome viscous friction $c_{\phi_{i}}$ and sustain oscillation. Equation (3a) represents the rotational dynamics of each pendulum: the first term is the moment of inertia multiplied by the angular acceleration, the second term corresponds to the torque exerted by the floating platform, the third term denotes the damping torque, and the fourth term is the gravitational restoring torque. The additional term $M_{D}$ accounts for the driving torque generated by the escapement mechanism. Equations (3b) and (3c) describe the translational motion of the platform in the *x* and *y* directions, respectively, obtained from the force balance of the entire system under the action of the pendulums. Finally, Equation (3d) is derived from the torque balance around the vertical axis and governs the rotational motion of the platform on the water surface.

We solved these equations numerically using the fourth-order Runge–Kutta method. The simulations employ the following constant parameters: $m_{1}=m_{2}=1.0$, $l_{1}=0.05$, $l_{2}=0.0501$, $x=y=\dot{x}=\dot{y}=\dot{\theta }=0$, M=5.0, $c_{x}=c_{y}=c_{\theta }=5.0$, $c_{\phi }=0.01$, b=2.0. Initial values for $\phi_{i}$ (i=1,2) are randomly selected within the range [0.5, 0.5]. The simulations successfully reproduce various dynamic behaviors, including in-phase synchronization, anti-phase synchronization, and synchronization with fixed phase differences. These numerical results demonstrate excellent agreement with experimental observations, validating the model’s capability to capture complex synchronization phenomena under varying initial conditions and parameter configurations.

## 5. Simulation Results

**Figure 6:**
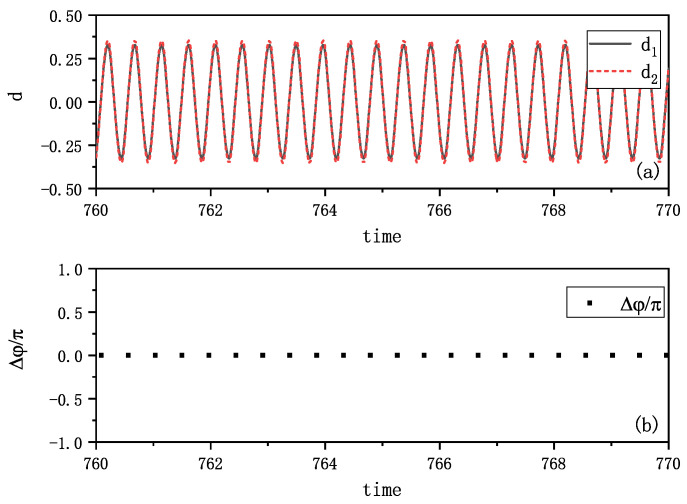
Simulation results of in-phase synchronization. (**a**) The distances between tips of metronomes and the balance position, i.e., the amplitudes of the vibrations $d_{i}=l^{\prime }*cos\phi_{i},\left(i=1,2\right)$ are plotted by time. The two distances totally vary in synchronization. (**b**) The phase differences of two metronomes($\Delta \varphi =\varphi_{1}-\varphi_{2}$) are calculated at the time when one metronome arrives at the balance position. They are zero as plotted. This kind of synchronization aligns with the phenomenon in Figure 2.

Our numerical simulations based on Equations (3) and (4) under varying initial conditions yielded results that closely match experimental observations. Figure 6 demonstrates remarkable agreement with the experimental data shown in Figure 2. The metronome tips exhibit complete synchronization in their oscillations, as illustrated in Figure 6a. We further analyzed the phase relationship by calculating the phase of one metronome using Equation (2) when the other metronome reaches its balance position (φ=π or −π), as shown in Figure 6b. The near-zero phase difference indicates simultaneous oscillations, consistent with the behavior observed in Figure 6a.

**Figure 7:**
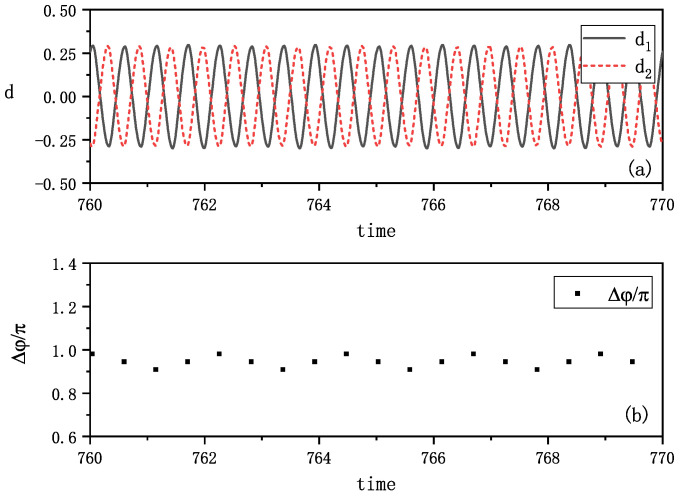
Simulation results of anti-phase synchronization. (**a**) The distances between tips of metronomes and the balance position $d_{i}\left(i=1,2\right)$ are plotted by time. The two distances vary oppositely and show an anti-phase synchronization. (**b**) The phase differences of two metronomes ($\delta \varphi =\varphi_{1}-\varphi_{2}$) are calculated at the time when one metronome arrives at the balance position. They vary at around π as plotted, which also means an anti-phase synchronization. This phenomenon aligns with the phenomenon that can be observed in Figure 3.

Under different initial conditions, we reproduced anti-phase synchronization, as shown in Figure 7. The amplitudes of the vibrations $d_{i}\left(i=1,2\right)$ plotted in Figure 7a clearly demonstrate opposing oscillations. The phase differences, calculated at instances when the metronomes reach their balance positions, are presented in Figure 7b. These differences consistently maintain values of π, confirming anti-phase synchronization and corresponding well with the experimental results shown in Figure 3.

**Figure 8:**
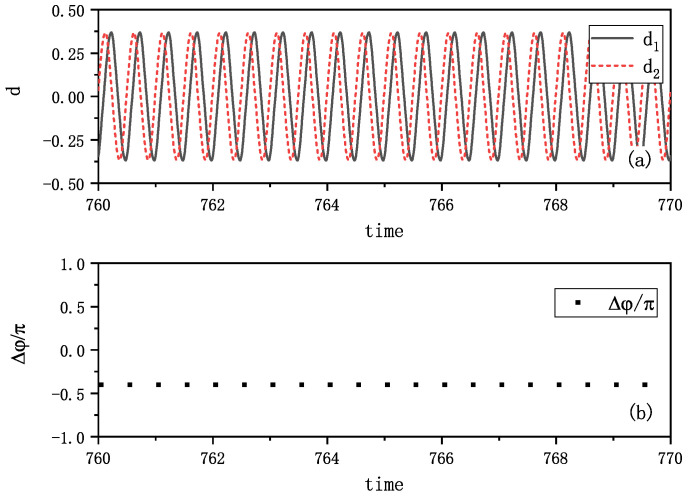
Simulation results of two metronomes oscillating with a fixed time delay. (**a**) The distances between the tips of metronomes and the balance position $d_{i}\left(i=1,2\right)$ are plotted by time. There is a time delay between the time when each metronome arrives its balance position. (**b**) The phase differences of two metronomes ($\delta \varphi =\varphi_{1}-\varphi_{2}$) are calculated at the time when one metronome arrives at the balance position. They are nearly 0.67π, and this means that the two metronomes oscillate with a fixed phase difference. This phenomenon is similar to the phenomenon observed in Figure 4.

A third synchronization mode emerged in our simulations, as depicted in Figure 8. Figure 8a shows the amplitudes of the vibrations $d_{i}\left(i=1,2\right)$, revealing that one metronome consistently leads the other with a fixed time delay. The phase differences, measured when one metronome reaches its balance position, converge to approximately 0.4π, as shown in Figure 8b. This behavior precisely mirrors the experimental observations presented in Figure 4.

**Figure 9:**
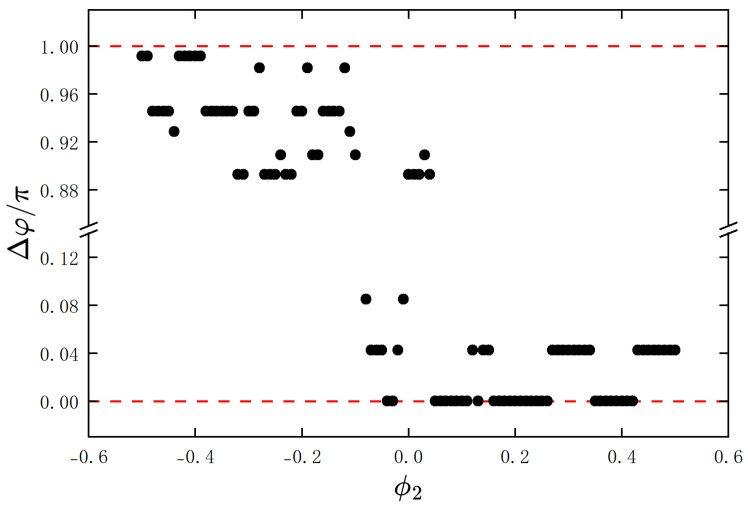
Simulation results of two metronomes’ different synchronization. We fixed the initial angular displacement of one metronome at $\theta_{1}=0.42$, and varied the initial angular displacement of another metronome from −0.5 to 0.5. The resulting final phase differences Δφ were calculated and are shown here. The two metronomes eventually reach in-phase synchronization, anti-phase synchronization, or synchronization with fixed phase differences. The red dashed line at Δφ/π=0 represents complete in-phase synchronization, and the red dashed line at Δφ/π=1 represents complete anti-phase synchronization.

In the numerical simulations, we also fixed the initial angular displacement of one metronome at ϕ=0.42, and varied the initial angular displacement of another metronome from −0.5 to 0.5. The resulting final phase differences Δφ were calculated, and are shown in Figure 9. As observed from the figure, depending on the initial conditions, the two metronomes eventually reach in-phase synchronization, anti-phase synchronization, or synchronization with fixed phase differences. Notably, the fixed phase differences in the phase-locked states exhibit distinct values. To provide a more comprehensive picture, we further explored the full parameter space by varying both $\phi_{1}$ and $\phi_{2}$. The corresponding two-dimensional phase diagram is presented in Figure S3 in the Supplementary Materials, where the global distribution of synchronization states can be clearly observed.

These numerical simulations provide strong validation of our experimental findings. The emergence of synchronization with fixed phase differences proves to be a robust phenomenon, with new synchronization states readily induced by changes in metronome oscillation directions. This means that a change in the vibrational direction of the metronome can affect their interaction, enabling the spontaneous emergence of a new steady state. In subsequent experiments, we will continue to investigate the effect of vibration direction on the different synchronization phenomena of the two.

The results clearly demonstrate that synchronization states are significantly influenced by initial conditions, particularly variations in pendulum lengths, showing excellent agreement with experimental observations.

## 6. Conclusions

In this study, we investigated the coupled dynamics of two metronomes mounted on a platform floating on water. In our configuration, the oscillation directions of the metronomes are arranged perpendicular to their connecting line, so that their interaction through the floating platform becomes two-dimensional rather than one-dimensional, which increases the complexity of the coupling. Through systematic analysis of video-recorded trajectories, we examined the metronomes’ movement patterns under various swing directions. Our extensive observations revealed three distinct synchronization modes: in-phase synchronization, anti-phase synchronization, and synchronization with fixed phase differences. Unlike the well-known cases of in-phase/anti-phase or classical phase-locking between oscillators, the fixed phase difference synchronization observed here is not characterized by a single unique phase offset. Instead, under the same system parameters, multiple stable phase differences can emerge depending on the initial conditions, revealing a qualitatively different phenomenon.

The frequencies of oscillations, the placement of the metronomes, and the material of the platform were varied to check the reliability of the new phenomenon. The synchronization with fixed phase differences appeared at higher oscillation frequencies. Moreover, if one of the metronomes was rotated by π, making the two metronomes vibrate back-to-back or face-to-face, the synchronization patterns were still maintained. In addition, when we changed the material of the platform to thermoplastic elastomer (TPE), all three synchronization types persisted. These results demonstrated that the third synchronization is robust and can be observed in a wide range of conditions.

Building upon existing models, we developed mechanical equations to describe the system and performed numerical simulations that successfully reproduced all three synchronization phenomena. The simulation results demonstrated excellent agreement with experimental observations. Furthermore, we investigated the role of platform material properties in the coupling strength between the metronomes, discovering that harder materials significantly enhanced synchronization efficiency. Our findings advance the understanding of coupled oscillator dynamics and offer potential insights for maritime engineering applications.

## Data Availability

The data presented in this study are available on request from the corresponding author.

## Supplementary Materials

The following supporting information can be downloaded at https://www.mdpi.com/article/10.3390/e27090908/s1: Figure S1: Experimental synchronization with a fixed time delay of the coupled metronomes; Figure S2: Experimental synchronization with a fixed time delay of the coupled metronomes; Figure S3: Simulation results of a two-dimensional phase diagram for the synchronization of two metronomes
