# Supplementary material for: Experimental and Numerical Study of Coupled Metronomes on a Floating Platform
Source: Entropy (Basel). 2025 Aug 27;27(9):908. doi: 10.3390/e27090908 (PMC12469052; doi:10.3390/e27090908)
Supplement: Supplementary file 1 [file entropy-27-00908-s001.zip › entropy-3833477-supplementary.pdf]

Supplementary materials

Fig.S1(a) shows another case that the two amplitudes of vibrations exhibiting snychronization with a fixed phase difference. The frequencies here is the same as that in Figs.2-4. We also calculated the phase differences $\Delta\varphi$ of two metronomes at the time when one metronome arrives at the balance position and found the phase difference converging to 0.58π (shown in Fig.S1(b)).

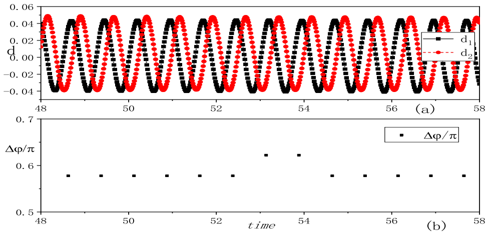

Fig. S1. Experimental synchronization with a fixed time delay of the coupled metronomes. (a)The distances between tips of metronomes and the balance positions $d_i(i = 1, 2)$, *i.e.*, the amplitudes of vibrations, are plotted via time goes. One metronome moves ahead of the other one, the time differences between the maximum swing position seems a fixed value. (b) The phase differences $\Delta\varphi$ of two metronomes are calculated at the time when one metronome arrives at the balance position. They are nearly 0.58π, and it means the two metronomes oscillate with a fixed phase differences.

We also varied the vibrational frequency to observe the synchronization. The metronomes are set achieving 100 ticks per minute, corresponding to a period of approximately 0.60 seconds. We again observed the phenomenon of fixed-phase-difference synchronization, as plotted in Fig.S2.

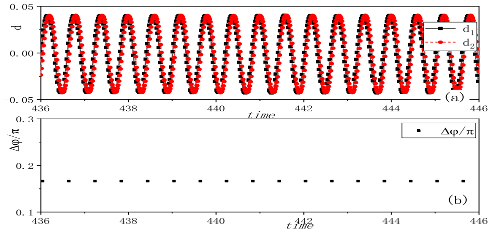

Fig. S2. Experimental synchronization with a fixed time delay of the coupled metronomes. The metronomes vibrate 100 ticks per minute here. (a) The distances between tips of metronomes and the balance positions $d_i(i = 1, 2)$, *i.e.*, the amplitudes of vibrations, are plotted via time goes. One metronome moves ahead of the other one, the time differences between the maximum swing position seems a fixed value. (b) The phase differences $\Delta\varphi$ of two metronomes are calculated at the time when one metronome arrives at the balance position. They are nearly 0.166π, showing the two metronomes oscillate with a fixed phase differences.

To further illustrate the influence of initial conditions on the synchronization behavior, we constructed a two-dimensional phase diagram as a function of the initial angular displacements ϕ_1 and ϕ_2 of the two metronomes. As shown in Fig. S3, the color scale represents the final phase difference $\Delta\varphi$ obtained after numerical evolution. The results clearly demonstrate that, depending on the initial states, the system evolves into in-phase synchronization, anti-phase synchronization, or synchronization with fixed phase differences. This supplementary figure provides a complementary perspective to Fig. 9 in the main text, highlighting the global structure of synchronization patterns in the phase space.

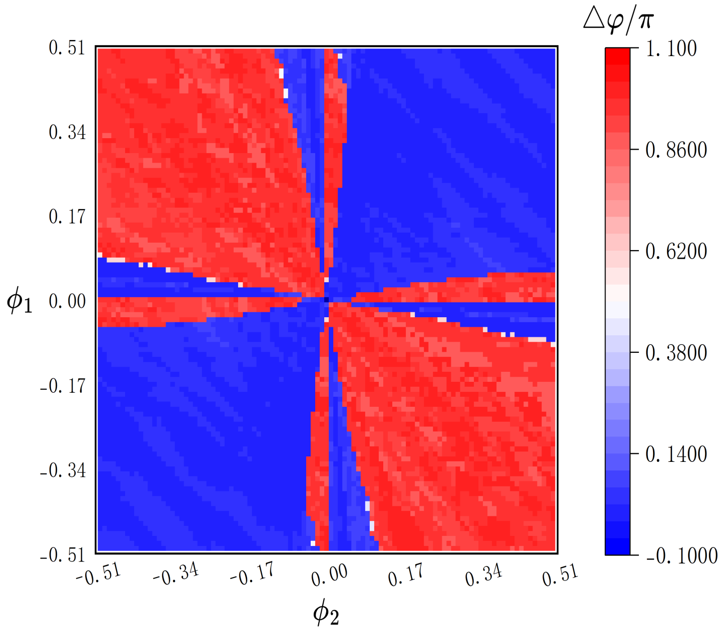

Fig. S3. Simulation results of a two-dimensional phase diagram for the synchronization of two metronomes. The horizontal axis represents the initial angular displacement of metronome 1 (ϕ_1), and the vertical axis represents that of metronome 2 (ϕ_2). The color scale indicates the final phase difference $\Delta\varphi$ after numerical evolution. Depending on the initial conditions, the two metronomes converge to in-phase synchronization, anti-phase synchronization, or synchronization with fixed phase differences.
